# Increased risk of atrial fibrillation in end-stage renal disease patients on dialysis: A nationwide, population-based study in Taiwan

**DOI:** 10.1097/MD.0000000000003933

**Published:** 2016-06-24

**Authors:** Cheng-Huang Shen, Cai-Mei Zheng, Kee-Thai Kiu, Hsin-An Chen, Chia-Chang Wu, Kuo-Cheng Lu, Yung-Ho Hsu, Yuh-Feng Lin, Yuan-Hung Wang

**Affiliations:** a Department of Urology, Ditmanson Medical Foundation Chiayi Christian Hospital, Chiayi; b Department of Health and Nutrition Biotechnology, Asia University, Taichung; c Graduate Institute of Clinical Medicine, College of Medicine; d Division of Nephrology, Department of Internal Medicine, School of Medicine, Taipei Medical University, Taipei; e Department of Nephrology, Shuang Ho Hospital, Taipei Medical University, New Taipei City; f Division of General Surgery, Department of Surgery, Shuang Ho Hospital, Taipei Medical University, Taipei; g Department of Urology, School of Medicine, Taipei Medical University, Taipei; h Department of Urology, Shuang Ho Hospital, Taipei Medical University; i Division of Nephrology, Department of Medicine, Cardinal-Tien Hospital, School of Medicine, Fu-Jen Catholic University; j Department of Medical Research, Shuang Ho Hospital, Taipei Medical University, New Taipei City, Taiwan.

**Keywords:** atrial fibrillation, end-stage renal disease, hemodialysis, peritoneal dialysis

## Abstract

End-stage renal disease (ESRD) patients commonly have a higher risk of developing cardiovascular diseases than general population. Chronic kidney disease is an independent risk factor for atrial fibrillation (AF); however, little is known about the AF risk among ESRD patients with various modalities of renal replacement therapy. We used the Taiwan National Health Insurance Research Database to determine the incident AF among peritoneal dialysis (PD) and hemodialysis (HD) patients in Taiwan.

Our ESRD cohort include Taiwan National Health Insurance Research Database, we identified 15,947 patients, who started renal replacement therapy between January 1, 2002 and December 31, 2003. From the same data source, 47,841 controls without ESRD (3 subjects for each patient) were identified randomly and frequency matched by gender, age (±1 year), and the year of the study patient's index date for ESRD between January 1, 2002 and December 31, 2003.

During the follow-up period (mean duration: 8–10 years), 3428 individuals developed the new-onset AF. The incidence rate ratios for AF were 2.07 (95% confidence interval [CI] = 1.93–2.23) and 1.78 (95% CI = 1.30–2.44) in HD and PD groups, respectively. After we adjusted for age, gender, and comorbidities, the hazard ratios for the AF risk were 1.46 (95% CI = 1.32–1.61) and 1.32 (95% CI = 1.00–1.83) in HD and PD groups, respectively. ESRD patients with a history of certain comorbidities including hypertension, diabetes mellitus, hyperlipidemia, coronary artery disease, heart failure, valvular heart disease, and chronic obstructive pulmonary disease (COPD) have significantly increased risks of AF.

This nationwide, population-based study suggests that incidence of AF is increased among dialysis ESRD patients. Furthermore, we have to pay more attention in clinical practice and long-term care for those ESRD patients with a history of certain comorbidities.

## 1 Introduction

With the progressive renal function impairment, chronic kidney disease patients usually experienced several comorbid conditions including cardiovascular disorders and at final end-stage renal disease (ESRD) stage, cardiovascular mortality accounts for about 50% of total mortality.^[1,2]^ Medical costs associated with these cardiovascular diseases are also increasing annually. Apart from coronary atherosclerosis, left ventricular hypertrophy (LVH), and dilation, congestive heart failure and cardiac arrhythmias are common contributors of cardiovascular mortality in these patients. Atrial fibrillation (AF) is the most common arrhythmia^[3]^; increasing prevalence is noted among dialysis patients (11%–27%) than age-matched general population (approximately 1%).^[4–7]^ Risk factors for AF have been investigated and renal function deterioration has been documented as independent risk factor for AF among many studies.^[8–10]^ Renal dysfunction also predicts AF-related stroke and related systemic thromboembolism.^[4,11,12]^

In general, ESRD patients undergo 1 of 3 modalities, hemodialysis (HD), peritoneal dialysis (PD), and renal transplantation. ESRD patients comprise 0.3% of the general population in Taiwan, and National Health Insurance (NHI) program covers NT$23 billion (occupy 6.18% of total medical expenditures) for dialysis treatment every year.^[13]^ Due to the shortage of donor kidneys, renal transplantation is less frequently performed. Therefore, more than 65,000 people receive a long-term renal replacement therapy in Taiwan; with approximately 91.5% undergo HD and 8.5% undergo PD.^[13]^ Incidence of new-onset AF in ESRD dialysis patients and risk factors associated with this has been studied by Liao et al^[14]^ recently. However, impact of dialysis on the incidence of AF in dialysis patients has not been determined. Thus, we conducted a nationwide, population-based study to evaluate the incidence and risks factors related with AF among Taiwanese HD and PD patients.

## 2 Methods

### 2.1 Data sources

The NHI program was implemented in March 1995 by the National Health Insurance Administration, Ministry of Health and Welfare, with a coverage rate of over 99% of the 23 million residents of Taiwan. The Longitudinal Health Insurance Database (LHID) was annually released by the National Health Insurance Administration for research purposes. The LHID contains the insured population registration files and medical claims data, including the information on basic demographic characteristics, inpatient and ambulatory care, diagnostic codes, medical expenditure, operations, prescriptions, examinations, and procedures. The International Classification of Diseases, 9th Revision, Clinical Modification (ICD-9-CM) was used for identifying diagnostic codes.

### 2.2 Study population

This study has a retrospective population-based cohort design. ESRD patients on a dialysis modality were selected from patients who were diagnosed with ESRD (ICD-9-CM 585) and started renal replacement therapy (HD or PD). All these ESRD patients had catastrophic illness registration cards. In Taiwan, ESRD patients who require a long-term dialysis modality are eligible to apply for a catastrophic illness registration card. The index date for ESRD patients was the date of their 1st diagnosis of new-onset ESRD. We excluded patients aged younger than 18 years or those aged older than 85 years. In addition, we excluded patients with a history of malignancy (ICD-9-CM 140–208) before the index date, patients with incomplete information on age and gender, and patients who did not receive a dialysis modality. Finally, a total of 17,213 patients, who started the renal replacement therapy between January 1, 2002 and December 31, 2003, were identified as the ESRD cohort.

Individuals without ESRD were selected randomly from the NHI beneficiaries registered in the LHID between January 1, 2002 and December 31, 2011 and were considered as eligible controls. For the controls, their 1st use of health care in this year served as their index health care use regardless of inpatient or ambulatory setting. We excluded patients with a history of malignancy before the index date, patients with incomplete information on age and gender, and patients aged younger than 18 years or older than 85 years. Finally, 47,841 eligible controls (at least 3 subjects for each ESRD patient) were identified randomly and frequency matched by gender, age (±1 year), and the year of the study patient's index date for ESRD. Both ESRD patients and controls were followed up from the index date to the onset of AF or until December 31, 2011.

### 2.3 Variable measurement

The age of participants was classified into 4 subgroups: <50, 50–60, 60–70, and ≥70 years. Geographic area was divided into Northern, Central, Southern, and Eastern Taiwan. AF patients were patients with 3 outpatient AF (ICD-9-CM 427.31) claims. In this study, atrial flutter (ICD-9-CM: 427.32) claims were excluded because, in general, the prevalence and mechanisms of atrial flutter differ from those of AF. Comorbidity was defined as a certain disease with at least 3 outpatient claims before the index date. These selected comorbidities included hypertension (ICD-9-CM 401–405), diabetes mellitus (ICD-9-CM 250), hyperlipidemia (ICD-9-CM 272), coronary artery disease (ICD-9-CM 410–414), hyperthyroidism (ICD-9-CM 242), heart failure (ICD-9-CM 428), valvular heart disease (ICD-9-CM 394, 396, 424, 746), LVH (ICD-9-CM 429.3), venous thromboembolic disease (ICD-9-CM 453), and chronic obstructive pulmonary disease (COPD) (ICD-9-CM 490–492, 494, 496). The duration from the implementation of dialysis to the newly onset AF was determined by estimating the time elapsed since the start of dialysis therapy and the onset of AF.

### 2.4 Statistical analysis

The chi-square test was used to compare the categorical variables among control, HD, and PD groups. The person-years of follow-up were calculated for each participant from the date of the index ambulatory care visit to the date of newly diagnosed AF, the date of death, or the end of the study. The incidence rate (per 1000 person-years) was calculated by dividing the number of incident AF by the person-years of follow-up as the denominator under the Poisson assumption. The Kaplan–Meier method and log-rank test were used to compare the risk of AF among the control, HD, and PD groups. The Cox proportional hazards model was used to estimate the hazard ratio (HR) and 95% confidence interval (CI). The adjusted HR was adjusted for age, gender, geographic area, and comorbidities, including hypertension, diabetes mellitus, hyperlipidemia, coronary artery disease, hyperthyroidism, heart failure, valvular heart disease, LVH, venous thromboembolic disease, or COPD. The SAS statistical package Version 9.3 (SAS Institute Inc., Cary, NC) was used for all statistical tests. Results with *P* < 0.05 were considered statistically significant.

### 2.5 Ethical approval

This study was reviewed and approved by the Taipei Medical University-Joint Institutional Review Board. As personal identification information was transformed and encrypted to protect the privacy of study participants, this study was exempted from full review by the Taipei Medical University-Joint Institutional Review Board (Approval No. 201211011).

## 3 Results

### 3.1 Patient demographics

We included 15,947 ESRD patients with dialysis who met our inclusion criteria, between January 1, 2002 and December 31, 2003, in the study group. Baseline characteristics of control and study groups are shown in Table 1. The mean ages were 60.8 ± 13.6, 61.3 ± 13.3, and 53.7 ± 15.0 years old in the control, HD, and PD groups, respectively. Regarding the duration from the implementation of dialysis to the newly onset AF, the mean ± standard deviation of the duration showed no significant difference between the HD group (2.89 ± 2.8) and the PD group (3.16 ± 2.9). Most ESRD patients also had the following comorbidities including hypertension, diabetes mellitus, dyslipidemia, coronary artery disease, hyperthyroidism, heart failure, valvular heart disease, LVH, venous thromboembolic disease, or COPD (Table 1).

**Table 1:**
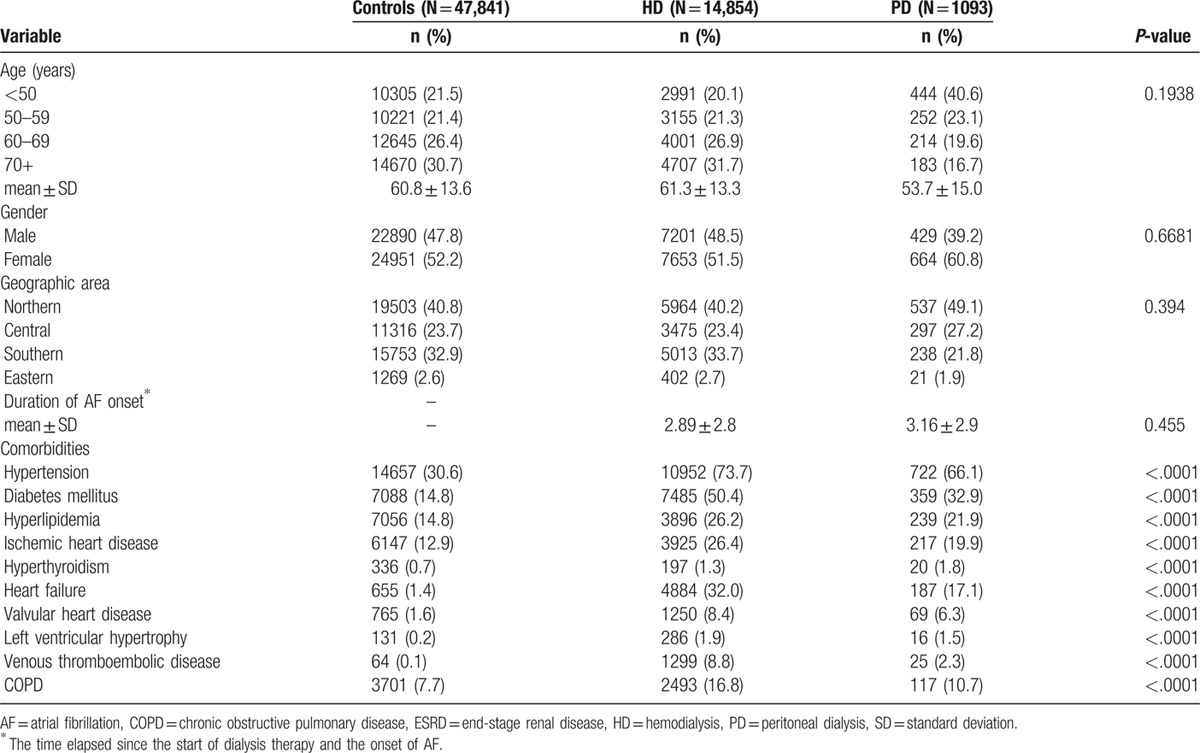
Basic characteristics and comorbidities of ESRD patients with dialysis modality.

### 3.2 Incident AF among PD, HD, and control groups

To estimate the incidence rate, we excluded patients who had a history of AF before the index date. During the mean follow-up period of 8 to 10 years, 3428 ESRD patients developed the new-onset AF (1318, 64, and 2046 subjects in the HD, PD, and control groups, respectively). The incidence rates of AF were 9.91, 6.42, and 4.86 per 1000 person-years in the HD, PD, and control groups, respectively. The incidence rate ratio for AF was significantly higher in the HD and PD groups than in controls, and was 2.07 (95% CI = 1.93–2.23) and 1.78 (95% CI = 1.30–2.44) in the HD and PD groups, respectively (Table 2). Compared with the controls, the risk of AF were 1.46 (95% CI = 1.32–1.61) and 1.32 (95% CI = 1.00–1.83) in HD and PD groups, respectively, after adjusting for age, gender, and comorbidities, including hypertension, diabetes mellitus, hyperlipidemia, coronary artery disease, hyperthyroidism, heart failure, valvular heart disease, LVH, venous thromboembolic disease, and COPD (model 2, Table 2).

**Table 2:**
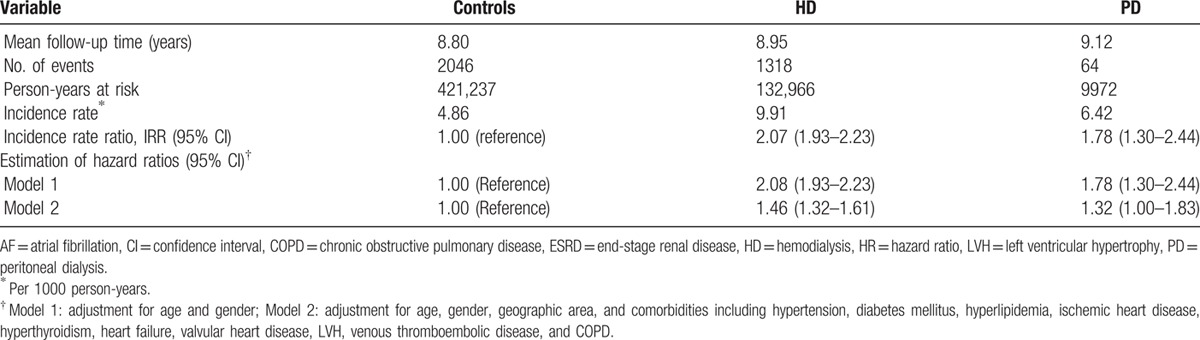
Incidence rate and HRs of AF for ESRD patients with dialysis modality.

### 3.3 Risks factors of new-onset AF in ESRD patients on different dialysis modalities

The adjusted HRs of AF (model 2) stratified by potential comorbidities for ESRD patients on different dialysis modalities are shown in Table 3. Compared with the control group, the significant HRs of 1.41 (95% CI = 1.22–1.62) and 1.51 (95% CI = 1.32–1.74) were found in the HD group for female and male ESRD patients, respectively. Among ESRD patients in different age subgroups, the significantly increased HRs of 6.36 (95% CI = 3.54–11.41), 2.65 (95% CI = 1.98–3.56), and 1.49 (95% CI = 1.33–1.68) were found in the HD group than those in the control group for <50 years, 50 to 59 years, and ≥60 years subgroups, respectively. Compared with the control group, those patients aged <50 years had a significantly increased risk of AF (HR = 5.18, 95% CI = 1.85–14.50) in the PD group.

**Table 3:**
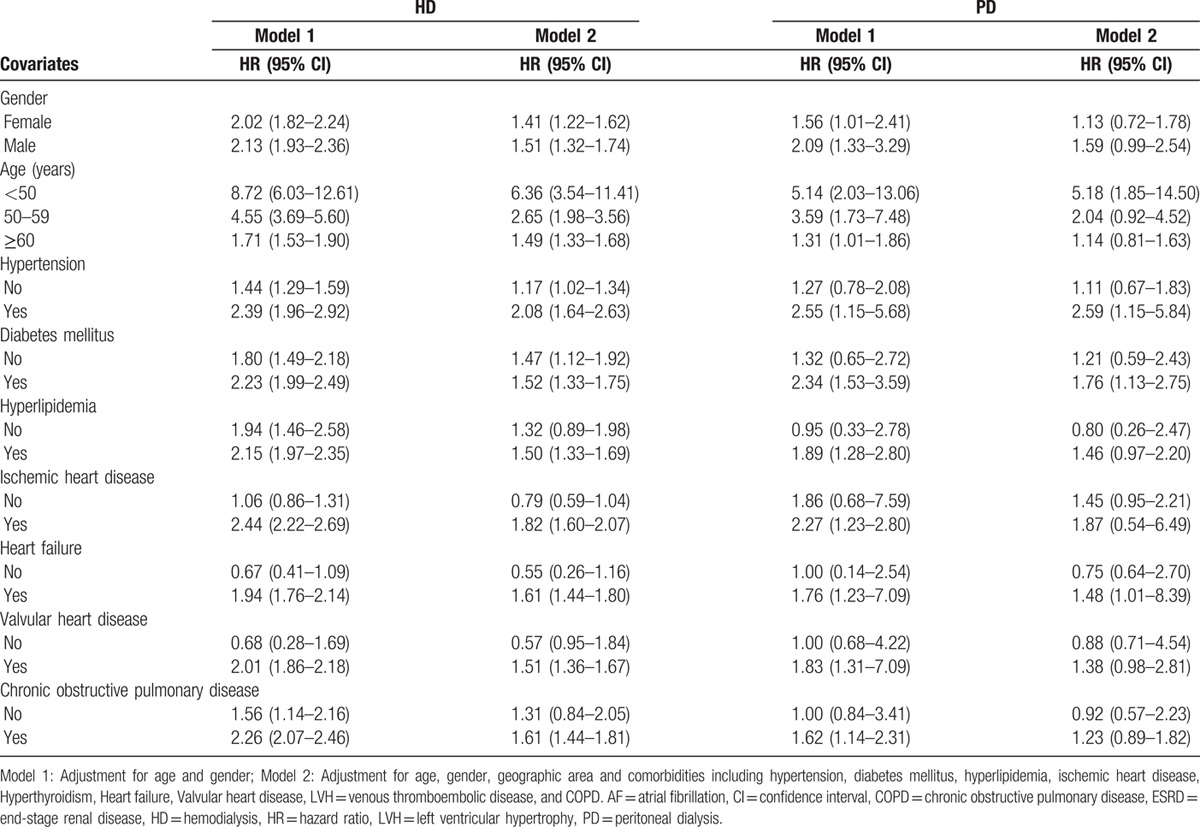
Risks of AF stratified by covariates for ESRD patients with dialysis modality.

The presence of certain comorbidities also significantly increased the risk of new-onset AF for the HD and PD groups, respectively. Those ESRD patients with hypertension had significantly higher risks of AF from the HR of 2.08 (95% CI = 1.64–2.63) in the HD group and from the HR of 2.59 (95% CI = 1.15–5.84) in the PD group. For ESRD patients with diabetes mellitus, significantly higher risks of AF were observed from the HR of 1.52 (95% CI = 1.33–1.75) in the HD group and from the HR of 1.76 (95% CI = 1.13–2.75) in the PD group. ESRD patients with dyslipidemia had a significantly higher risk of AF from the HR of 1.50 (95% CI = 1.33–1.69) in the HD group. ESRD patients with coronary artery disease in the HD group had an adjusted HR of 1.82 (95% CI = 1.60–2.07). Those ESRD patients with heart failure had significantly higher risks of AF from the HR of 1.61 (95% CI = 1.44–1.80) in the HD group and from the HR of 1.48 (95% CI = 1.01–8.39) in the PD group. In addition, ESRD patients with valvular heart disease had a significantly higher risk of AF (HR = 1.51, 95% CI = 1.36–1.67) in the HD group. Regarding those ESRD patients with COPD, a significantly higher risk of AF (HR = 1.61, 95% CI = 1.44–1.81) was identified for the HD group.

## 4 Discussion

We investigated the risk of new-onset AF among ESRD patients receiving dialysis using National Health Insurance Research Database. We stratified the patients according to different age groups, gender, geographic area, duration of AF onset, and comorbid conditions. The mean ages in the control, HD, and PD groups were not different significantly. ESRD patients had significantly increased comorbid conditions compared with control group including hypertension, diabetes mellitus, dyslipidemia, coronary artery disease, hyperthyroidism, heart failure, etc. in both HD and PD groups (Table 1). Study findings can be summarized as follows (Tables 2 and 3): the incidence rates of AF were 9.91, 6.42, and 4.86 per 1000 person-years in HD, PD, and control groups, respectively; after adjusting age, sex, and comorbid conditions, adjusted HRs for the risk of AF were 1.46 (95% CI = 1.32–1.61) in HD and 1.32 (95% CI = 1.00–1.83) in PD groups, respectively; and the presence of comorbid conditions significantly increased the risk of new-onset AF in dialysis population. To our knowledge, this is the 1st study to determine the incidence of AF among ESRD patients undergoing different dialysis modality in Taiwan.

Several studies have reported the incidence of AF in ESRD patients varying from 1.0 to 14.8 per 100 person-years.^[7,15–19]^ Goldstein et al^[19]^ reported an AF incidence of 148/1000 person-years in a Medicare database with a total of 258,605 elderly ESRD patients. Wizemann et al^[18]^ using the data from international Dialysis Outcomes and Practice Patterns Study to analyzed the incidence, prevalence, and outcomes of AF among HD patients. Age, gender, racial, and geographical factors might considerably influence the incidence of AF among different population studies. South or Southeast Asians including Chinese population undergoing maintenance dialysis therapy experience a lower tolerability to fluid expansion, more cardiovascular events,^[20–22]^ and cardiovascular mortality^[23]^ than African American population. From our study, we found the incidence rates of AF are 9.91 and 6.42 per 1000 person-years in HD and PD groups, respectively.

We found significantly higher events of AF among dialysis patients in those undergoing either HD or PD compared with control group. A study conducted by Abbott et al,^[24]^ their results reported from the United States Renal Data System and the Dialysis Morbidity and Mortality Study Wave 2 database revealed that those undergoing HD had a significantly increased risk of AF than those with PD.^[24]^ Nonphysiological nature of HD modality might explain more incidents of AF among this population. The occurrence of symptomatic atrial arrhythmias in the last hours of HD^[15]^ might relate with development of AF in HD patients. Further, the progressive cardiac enlargement, especially LVH, occurs during the early years among HD patients^[25]^ and LVH progresses independent of adequate blood pressure control.^[26]^ In present study, we also found PD patients had higher incidence of AF than control patients. Although PD is more likely physiologic than HD model, some studies also revealed prevalent LVH among chronic PD patients.^[27,28]^ Wang et al^[29]^ revealed 30% of PD patients had cardiac valve calcification; vascular and valvular calcifications associated with increasing risk of valvular or nonvalvular AF.^[30]^

We interestingly found that HRs for the risk of AF were even higher in dialysis patients after adjusting age, gender, and comorbid conditions. Apart from traditional risk factors, non-Black race, routine use of calcium containing phosphate binders and higher dialysate calcium, renal failure associated bone, and mineral disorders might be unique factors explained the higher risk of new onset AF in this population.

Further, the presence of certain comorbidities including hypertension, diabetes, dyslipidemia, coronary artery disease, heart failure, valvular heart disease, and COPD also significantly increased the risk of new-onset AF for the HD and PD groups. Valvular calcification is a common complication of dialysis patients and occurs as a result of dysregulation of calcium and phosphate metabolism. Calcification of the cardiac valve leaflets can change the mechanical properties of the cardiac tissue^[31]^ and results in development of AF. With loss of residual renal function, most of these patients experienced chronic volume overload, LVH, and repeated heart failure, all of which are found to increased incident AF in our patients. Respiratory and renal diseases are frequently coexisting, and most dialysis patients experienced intradialytic hypoxemia. Abnormal pulmonary functions, dialytic, and pulmonary hypertension related hemodynamic changes can lead to the development of AF. Medications used for COPD prevention and exacerbation, for example, beta-adrenergic agonist and theophylline might associated with development of AF.^[32]^ Some studies also point out the importance of these risk factors in chronic dialysis patients.^[33–35]^ Similarly to the general population, a high incidence of stroke in AF might lead to high mortality in this population.^[36]^

This study has several limitations. First, we did not include individual behaviors that related with AF including smoking habits, alcohol intake, and physical activity, etc. and clinical data including body mass index, severity of comorbidities, residual renal function, and actual blood pressure values were lacking. Second, we relied on the ICD-9-CM diagnosis codes of AF and comorbidities associated with AF; and echocardiographic parameters such as left atrial dimension, LVH, and valvular status were also lacking. However, recent clinical diagnosis of AF is largely based on electrocardiogram, we believed most of our study could represent most of the AF population. Finally, our study lacked specific data on dialysis vintage, fluid management during dialysis sessions, and intradialytic hemodynamic change, and we did not analyzed specific medications which might influence AF in this population.

In conclusion, increased risk of AF is noted among ESRD patients on dialysis especially with certain comorbidities. Although we can only show a relationship, not causality, between dialysis and incident AF, this relationship is clinically crucial since it can help physicians to pay more attention on AF related morbidity and mortality among dialysis patients.

## Acknowledgments

The authors thank Shuang Ho Hospital, Taipei Medical University (102TMU-SHH-10), Chiayi Christian Hospital (R102-7), and the Health Promotion Administration, Ministry of Health and Welfare (DOH102-HP-1103) for the support.
